# Practical bioinformatics pipelines for single-cell RNA-seq data analysis

**DOI:** 10.52601/bpr.2022.210041

**Published:** 2022-06-30

**Authors:** Jiangping He, Lihui Lin, Jiekai Chen

**Affiliations:** 1 Center for Cell Lineage and Atlas (CCLA), Bioland Laboratory (Guangzhou Regenerative Medicine and Health Guangdong Laboratory), Guangzhou 510320, China; 2 Key Laboratory of Regenerative Biology of the Chinese Academy of Sciences and Guangdong Provincial Key Laboratory of Stem Cell and Regenerative Medicine, Guangzhou Institutes of Biomedicine and Health, Chinese Academy of Sciences, Guangzhou 510530, China

**Keywords:** Single-cell RNA sequencing (scRNA-seq), scRNA-seq analysis, Practical bioinformatics pipeline

## Abstract

Single-cell RNA sequencing (scRNA-seq) is a revolutionary tool to explore cells. With an increasing number of scRNA-seq data analysis tools that have been developed, it is challenging for users to choose and compare their performance. Here, we present an overview of the workflow for computational analysis of scRNA-seq data. We detail the steps of a typical scRNA-seq analysis, including experimental design, pre-processing and quality control, feature selection, dimensionality reduction, cell clustering and annotation, and downstream analysis including batch correction, trajectory inference and cell–cell communication. We provide guidelines according to our best practice. This review will be helpful for the experimentalists interested in analyzing their data, and will aid the users seeking to update their analysis pipelines.

## INTRODUCTION

**Figure 1:**
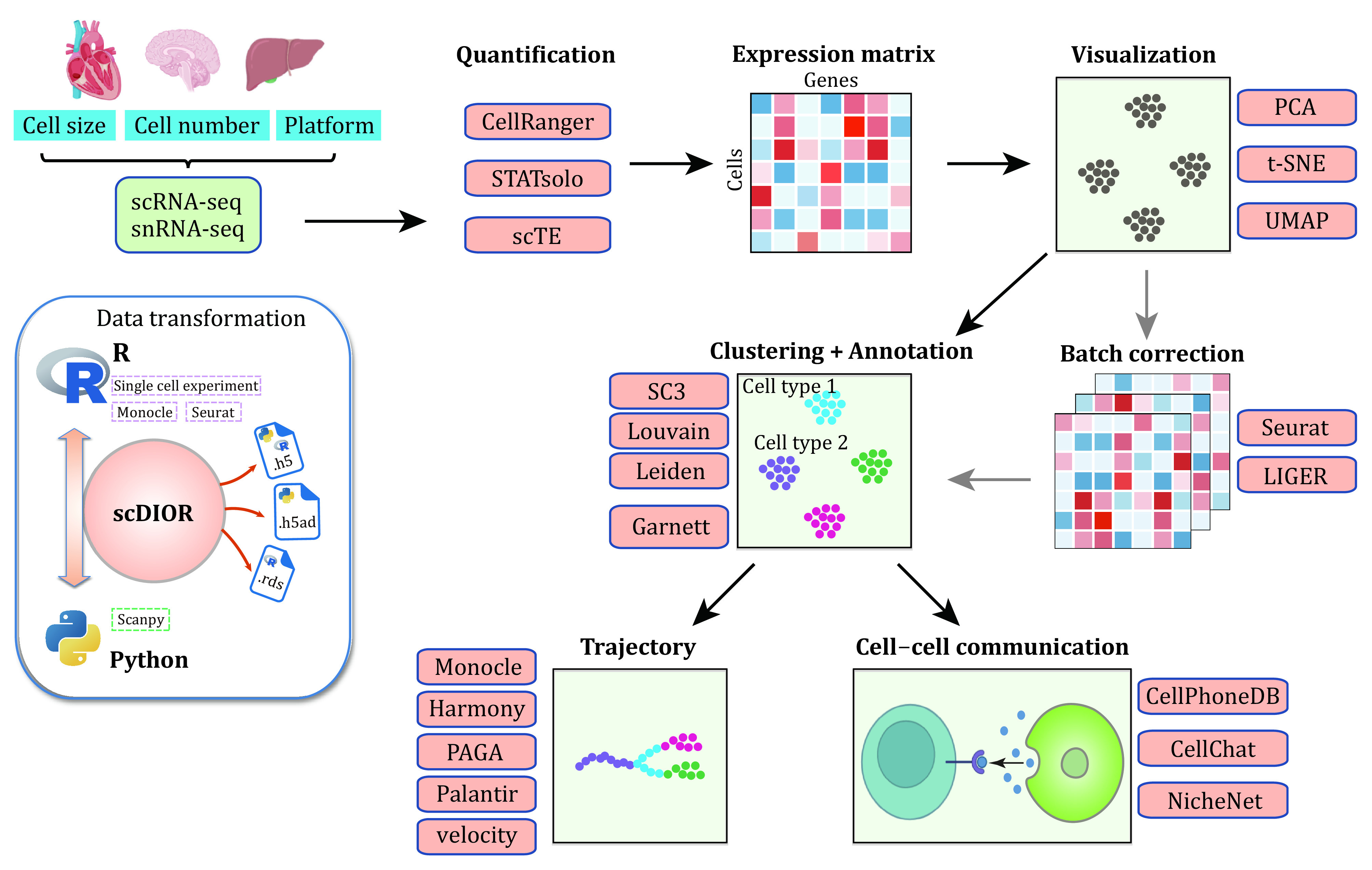
Overview of scRNA-seq data analysis workflow. Typically, the researchers must first consider which platform of single-cell are needed as each has its benefits and limitations. After sequencing, the expression matrices can be obtained by quantification tools. Sometimes, researchers need to combine expression matrices using batch correction methods. Next, the data are visualized by dimensionality reduction, and clustered and annotated for biological interpretation, or ordered the cells along a predicted trajectory in pseudotime, or inferring for cell-cell communication. Suggested tools are listed in the colored box

ScRNA-seq has developed as a powerful tool to characterize complex tissue and answer the question that cannot be addressed by bulk RNA-seq. Many new single-cell technologies have been developed to discover missing observations, including the methods for measurement of the single-cell genome sequence, chromatin accessibility, DNA methylation, histone modification, transcription factor binding and chromatin conformation (Cao* et al.* 2018; Hainer* et al.* 2019; Stuart and Satija 2019; Wang* et al.* 2019). ScRNA-seq is the most commonly used technique in the community that has significantly advanced our knowledge of the biological process. However, due to the sparse and high dimensional nature of scRNA-seq data, scRNA-seq data analysis is still challenging. The scRNA-seq technology, its application and analysis methods have been previously reviewed (Andrews* et al.* 2021; Bacher and Kendziorski 2016; Luecken and Theis 2019; Paik* et al.* 2020; Papalexi and Satija 2018; Potter 2018). In this review, we focus particularly on the bioinformatics analysis pipeline (Fig. 1) and spotlight the “best” pipelines according to our practice.

## EXPERIMENTAL DESIGN FOR SCRNA-SEQ

In the past decades, a variety of single-cell approaches have been developed for cell capture and RNA amplification, each has its unique advantages and disadvantages (Lafzi* et al.* 2018; Paik* et al.* 2020; Papalexi and Satija 2018). According to the single-cell isolation and capture strategy, currently, scRNA-seq techniques can be categorized into two main approaches, the plate- or microfluidic-based methods and droplet-based methods. Plate-based protocols use the fluorescence-activated cell sorting (FACS) to isolate the individual cells. Automated microfluidic-based platforms, such as the Fluidigm C1, isolate and capture the single cells with parallel microfluidic channels. The plate- and microfluidic-based methods are similar, and are often limited in the throughput with ~50 to ~500 cells per analysis. The key potential benefit of those platforms is that they generally have high sensitivity with reliably quantifying up to ~10,000 genes per cell. Droplet-based methods barcoded single cells and tagged each transcript with unique molecular identifiers (UMI) in individual oil droplets, thus substantially reducing the time and cost needed per analysis, and massively increasing the throughput to up to ~10,000 cells per run. However, owing to technical limitations, typically detect only 1000–3000 genes per cell, which is just a small part of genes actually expressed. The genes that are expressed but the transcripts are not detected due to the technical issues termed dropouts.

The experimental design considerations for scRNA-seq have been previously reviewed (Baran-Gale* et al.* 2018; Lafzi* et al.* 2018). Several factors need to be considered before choosing a scRNA-seq method. First, is the number of cells that need to be sequenced per experiment. This is highly dependent on the heterogeneity of all cells in the sample, and dependent on the proportion of a particular cell type you expected within the sample, according to the previous knowledge. The satija lab provides an online tool (https://satijalab.org/howmanycells/) for estimating how many cells need to sample according to the cell diversity. In case no prior knowledge is available about the heterogeneity of the cell population, a practical solution is to perform the study with a high cell number and lower sequencing depth, and then perform pre-purification of the interested cells by FACS with in-depth sequencing. Another factor that needs to be considered is the cell size. Each platform has its limitation. Upon technical demands, cell size is one factor. Smaller cells (less than 25 μm in diameter) are generally easier to be processed with minimal damage compared to the larger or irregular-shaped cells, *e*.*g*., adult cardiomyocytes and neurons (Paik* et al.* 2020). Thus, single nuclei RNA-seq (snRNA-seq) arises as an alternative approach (Grindberg* et al.* 2013; Lacar* et al.* 2016; Litvinukova* et al.* 2020). Third, avoiding technical biases, even though increasing methods are developed to remove the technical biases for data analysis, it is still challenging to distinguish the technical noise from the real biological variance. It is important to design a balanced experiment that limits confounding factors.

## PRE-PROCESSING AND QUANTIFICATION

Once the sequencing reads are obtained, quality control (QC) should be performed for the raw reads the same as bulk RNA-seq. FastQC (https://www.bioinformatics.babraham.ac.uk/projects/fastqc/) is one of the popular tools for checking read quality within an individual sample, which inspects several quality metrics and provides reports with informative visualizations. FastQC reports and visualizes information on base quality, GC content, adapter content and also the presence of ambiguous bases, and over-represented sequences, the FastQC website discusses these and other issues in detail. Trimming is useful to remove adapters and cut reads in different ways based on quality, which may improve the reads mapping. Trimmomatic (Bolger* et al.* 2014), Trim Galore (https://www.bioinformatics.babraham.ac.uk/projects/trim_galore/) and cutadapt (Martin 2011) are all popular tools for cutting the reads based on quality score or adapter sequence.

With the quality reads in hand, the expression of each gene in individual cells needs to be estimated. For non-UMI and non-barcode datasets, expression can be obtained with traditional bulk RNA-seq quantification tools such as RSEM (Li and Dewey 2011), STAR (Dobin* et al.* 2013) and HTSeq (Anders* et al.* 2015), and the downstream analysis have also successfully adopted from bulk RNA-seq pipelines. If UMI- and barcode-tagged data are available, counts can be obtained by CellRanger (Zheng* et al.* 2017) or STARsolo (Kaminow* et al.* 2021). In our practices, STARsolo is 10 times faster than the CellRanger and outputs nearly identical results (He* et al.* 2021; Kaminow* et al.* 2021). These approaches map sequencing reads to a reference genome or transcriptome index, and typically report gene expression as raw counts. Nearly all quantification tools are typically gene-centric. Recently, we developed a single-cell transposable elements (TEs) expression processing pipeline, scTE (He* et al.* 2021), which quantifies the expression of genes and TEs in the same single-cell, and demonstrated TEs are useful addenda to the gene information, and in some cases are the major source of information of their own in a variety of systems and human disease.

## QUALITY CONTROL

scRNA-seq data quality control can be split into cell QC and gene QC. For cell QC, the first step is to exclude all the cell barcodes that are unlikely to represent intact individual cells, those are generally dead cell debris and free-floating RNAs. The most straightforward approach for assessing the quality of a cell is to calculate the number of molecule counts (UMIs), the number of expressed genes, the total detected counts, and the proportion of RNA from mitochondrial genes. Cells with high proportions of mitochondrial derived reads, a low proportion of nuclear RNAs are often damaged or dying cells, but it should be noted that a high fraction of mitochondrial RNAs can also be biological signals that indicate elevated respiration, such as in the cardiomyocytes. In our practice, cells with less than 1000 UMIs and less than 500 genes detected were filtered out. Cells with more than 20% fractions of mitochondrial counts were also discarded.

In contrast, cells with unexpectedly high counts and a too large number of expressed genes may represent doublets (or multiplets), that cell barcodes might correspond to more than one cell. Removing doublets is important for the high throughput scRNA-seq method, of which often ~5% of cell barcodes are tagging multiple cells (Wolock* et al.* 2019), and recent results suggest that up to ~20% in the droplet-based 10x Chromium scATAC-seq assay (Lareau* et al.* 2020). Unfortunately, neither of these approaches can effectively distinguish real single-cell from doublets. Doublets often harbor “hybrid” expression features, special tools such as scrublet (Wolock* et al.* 2019), DoubletFinder (McGinnis* et al.* 2019) and scds (Bais and Kostka 2020) are developed to infer potential doublets from the dataset itself.

In addition to QC of the cells, QC steps can also be performed at the gene level. Raw counts often include over 20,000 to 50,000 genes, which depends on the reference index. This number can be dramatically reduced by filtering out the genes that is not expressed or only expressed in extremely few cells, which are not informative of cell identity and cellular heterogeneity. This is helpful to reduce the computational time and memory cost for the downstream analysis. It is important to choose a proper threshold for filtration, a guideline for this is to use the minimum cell cluster size of interest with consideration of dropouts. For example, filtering out the genes that are expressed in less than 20 cells may make it difficult to detect the cell clusters with fewer than 20 cells. If the datasets have high dropout rates, this threshold may even difficult to find larger clusters. In short, this threshold depends on the cell number of interest and should be scaled with the total cell number of the datasets. Nevertheless, in our practices, we did not recommend QC for genes unless the datasets are too big out of the limitation of computational resources of the computing server. First, as discussed above, it should be careful for setting the threshold. Besides, filtering genes makes it difficult to perform comparable analysis for the datasets from different studies, as the genes that have been filtered out in this study may be informative for another one.

## NORMALIZATION

Most quantification tools generally output the raw counts’ matrix, counts are representative of the molecules that are successfully captured, reverse transcribed and sequenced in the scRNA-seq experiment. While the number of useful reads obtained from a scRNA-seq experiment varies between cells, and this effect is pronounced for scRNA-seq experiment owing to the biological and technical factors, this difference must be corrected. The most commonly used normalization method accounts for depth scaling. As not all RNA molecules in a cell are not captured, some scRNA-seq experiments use spike-ins, the synthetic exogenous molecules at known content, to improve the global scaling factors, but the use of spike-ins in scRNA-seq is not routine yet due to the changes in generating high-quality and representative spike-ins. An increasing number of normalization methods have developed, and the scran package (Lun* et al.* 2016) has been shown to perform better than other tested normalization methods for the downstream analysis (Buttner* et al.* 2019), while another study argues that different normalization method performs optimally for different datasets and claim that their scone tool can be used to evaluate the impact of the statistical design and select an appropriate normalization for a given study (Cole* et al.* 2019). After normalization, the data matrices are typically log(x+1)-transformed for the downstream analysis.

## FEATURE SELECTION

scRNA-seq faces the challenge of dropout, data only captures a small fraction of the transcriptome of each cell. The gene expression could vary across all cells due to the technique noise, especially for the genes with a low amount of RNA (Brennecke* et al.* 2013). The variance accounting for technical noise will disturb the downstream analysis, such as dimensional reduction and clustering. It is necessary to select the genes with biological variance over technical variance, namely highly variable genes (HVGs). HVGs can be identified by fitting the variance-mean of each gene by a generalized linear model (GLM) (Brennecke* et al.* 2013; Stuart* et al.* 2019). A high level of variance (exceeding the specified threshold) will indicate genes important in explaining heterogeneity within the cell population under study. Standard deviation (std) or squared coefficient of variance (CV2) are commonly used to estimate gene variance, however, such an index prefers the genes with larger mean expression value. The genes only expressed in rare cells may be missed by such strategies. GiniClust (Jiang* et al.* 2016) first note that cell clustering is dependent on the selection of genes. GiniClust introduces Fano factor and Gini index to identify rare cell type expressed genes. Users should select the best feature selection strategy according to their research purpose.

## DIMENSIONALITY REDUCTION AND VISUALIZATION

The high-dimensional nature of scRNA-seq dataset, in which a single gene represents a single dimension. For example, if 3,000 genes are selected as HVGs for the downstream analysis, it has 3,000 dimensions and must be reduced into 2 or 3 dimensions for interpretable visualization and analysis. To date, the dimensionality reduction techniques can be divided into linear and non-linear algorithms. Principal component analysis (PCA), a linear transformation approach that perseveres Euclidean distance between cells, is the most commonly used algorithm for bulk RNA-seq data dimension reduction, and also have been successfully adopted for the low throughput but in-depth scRNA-seq data analysis (Goke* et al.* 2015; Mohammed* et al.* 2017). While PCA cannot effectively capture cellular relationships due to high level dropouts and other technical noise, it often performs poorly for the direct visualization of high throughput but low depth scRNA-seq datasets. However, as PCA can be calculated efficiently even for large datasets, it is the most commonly used as a pre-processing step for non-linear dimensionality reduction algorithms, and it is the basis of many clustering and trajectory inference analysis tools. Non-linear dimensionality reduction algorithms, such as t-distributed stochastic neighbor embedding (t-SNE) (van der Maaten and Hinton 2008) have been very popular in the community, but recent uniform manifold approximation and projection (UMAP) (Becht* et al.* 2018) algorithm has been shown to better represent the topology of the data. Besides, some publicly available tools, such as Seurat (Butler* et al.* 2018) and Scanpy (Wolf* et al.* 2018), allow projection and visualization of both t-SNE and UMAP from custom defined PCAs.

## BATCH CORRECTION

The single-cell projects such as Human Cell Atlas (Regev* et al.* 2017) aim to define all human cell types in terms of distinctive molecular profiles (such as gene expression profiles) and to connect this information with classical cellular descriptions (such as location and morphology) through an international collaborative effort. A comprehensive reference map of the molecular state of cells in tissues would propel the systematic study of physiological states, developmental trajectories, regulatory networks and interactions of cells, and also provide a framework for understanding cellular dysregulation in disease. However, biological data are affected by the conditions of the measuring experiments, such as different cell dissociation and handling protocols, library-preparation technologies and/or sequencing platforms (Haghverdi* et al.* 2018). The term batch effect describes a situation where batches of the data significantly differ in distribution, due to irrelevant instrument-related factors (Leek* et al.* 2010). The systematic error introduced by batch effects may obfuscate the signal of interest. The Batch effect correction in single-cell RNA-seq data is a task to identify and remove confounding factors between batches. However, batch effects may be confounded with the biological covariate of interest, such as drug-treated condition or developmental process. It is a great challenge to remove batch effects and retain real biological heterogeneity at the same time.

Recently, an increasing number of algorithms are proposed. scMerge (Lin* et al.* 2019) firstly identifies the single-cell stably expressed genes (scSEGs) as “negative controls” for estimating the unwanted factors, which are then used to correct batch effect by fastRUVIII algorithm (Risso* et al.* 2014), an RNA-seq normalization method by factor analysis of control genes or samples. ZINB-WaVE (Risso* et al.* 2018) uses a negative binomial (ZINB) model accounting for zero inflation (dropouts), over-dispersion, and the count nature of the data. ZINB-WaVE is an extension of the RUV model, which includes observed and unobserved sample-level covariates and enables normalization for batch effects. Although ZINB-WaVE models sophisticated single-cell data, it fails to handle large scale data sets with tens of thousands of cells. Matching mutual nearest neighbors (MNN) (Haghverdi* et al.* 2018) identifies cells that have mutually similar expression profiles between different experimental batches or replicates. The authors inferred that any differences between these cells in the high-dimensional gene expression space are driven by batch effects and do not represent the underlying biology of interest. The systemic difference between these cells is extracted by the algorithm and used to correct the batch effects. The idea of MNN has a great impact on the latter algorithms. MNN was originally designed to batch correction for pairwise datasets, if there are more than one datasets to be integrated, all the datasets will be projected to one user selected dataset sequentially. The result is inconsequent if there are no common cell states for some pair of datasets. Scanorama (Hie* et al.* 2019) integrates data from heterogeneous scRNA-seq experiments by finding common cell types among all pairs of datasets. The mutually linked cells between two datasets are kept. This procedure excludes spurious links from the neighbor searching. Conos (Barkas* et al.* 2019) also apply MNN to integrate multiple datasets in low-dimensional space. Instead of correction of gene expression, Conos aims to construct a joint graph of cells. Since the MNNs were detected using L2 normalized gene expression, significant differences between batches may obscure the identification of MNNs. To overcome this, Seurat v2 (Butler* et al.* 2018) uses canonical correlation analysis (CCA) to integrate datasets, which projects the cells into the most correlated components between two data sets. The rare cells that cannot be explained by CCA are flagged for further analysis. A nonlinear “warping” algorithm is then used to align the data sets into a conserved low-dimensional space. Although CCA can identify shared biological markers and conserved gene correlation patterns, the different cell types should not be aligned together. Therefore, Seurat v3 integrates CCA and MNN and introduces “anchors”, cell pairs that encode the cellular relationships across datasets. The anchors are important to determine the correction vector, which is used to correct the gene expression profile. LIGER (Liu* et al.* 2020a) takes as input multiple datasets of batches and learns a low-dimensional space using integrative non-negative matrix factorization. LIGER enables the identification of shared cell types across batches, as well as dataset-specific features, offering a unified analysis of heterogeneous single-cell datasets. Besides learning a low dimensional space, Harmony (Korsunsky* et al.* 2019) introduced an iterative procedure to soft cluster and correct clusters center of cells. After clustering, each dataset has cluster-specific centroids that are used to compute cluster-specific linear correction factors. Based on the linear nature of the clustering and correction algorithm, Harmony scales with large datasets. Although the batch effects can be successfully corrected for some particular scenarios, most methods presume that datasets share the same cell type. The uncorrected anchors identified by some algorithms between two datasets will lead to erroneous correction.

Unfortunately, these algorithms may not be able to correctly adjust the batch effects for non-shared cell groups due to the non-linear transformation. Recently, Reference Principal Component Integration (RPCI) (Liu* et al.* 2021) was proposed to integrate multiple datasets without the presumption of inter-sample similarity and does not rely on shared cell types. RPCI uses a global reference gene eigenvector to decompose all datasets and project all cells into RPCI space. Note that the linear RPCI space captures the information of gene expression differences among datasets, which retained the dissimilarities in cell groups. The batch effect removal algorithms are becoming powerful tools for single cell atlas, which make it possible to integrate and compare data from different laboratories, conditions, and cells* in vitro* and *in vivo*. The algorithms need to be optimized to accommodate more complex scenes and reduce computing consumption as more and more cells can be sequencing in the future. Notably, this step is optional if did not observe the obvious batch effect.

## CLUSTER ANALYSIS AND ANNOTATION

The key advantage of scRNA-seq over traditional bulk RNA-seq is the characterization of the heterogeneity of cell populations at the single-cell level. In biological terms, cell populations in a given sample may represent many different cell types, such as the heart contain cardiomyocytes, fibroblasts, endothelial cells and immune cells. But they may also represent a different state of the same cell type, such as the stress response and immune response to infection, or the cell in the normal and diseased state. Cell cluster is central for most scRNA-seq data analysis, as it unbiased identifies groups of cells based on expression profiles. Cells with similar expression patterns are considered as the same cell types or states, while other cells are classified as distinct cell types. However, due to the sparse and high dimensional nature of scRNA-seq data, identifying cell groups based on their transcriptome is a challenging task.

Cell clustering generally after feature selection and dimensionality reduction. A number of clustering strategies for scRNA-seq data analysis have been established. Supervised clustering refers to approaches that classify cells based on prior known paradigm, such as cell type specific marker genes. Such as MetaNeighbor (Crow* et al.* 2018) allow users to assess how well cell-type-specific transcriptional profiles replicate across datasets, CellAssign (Zhang* et al.* 2019) and Garnett (Pliner* et al.* 2019) leverage prior knowledge of cell-type marker genes to annotate single-cell RNA sequencing data into predefined or *de novo* cell types. Conversely, unsupervised clustering refers to approaches that classify cell with the data itself without any intervention from prior knowledge or other datasets. One example is the widely used k-means algorithm, which iteratively tests the closest k-cluster center to which each cell is assigned. In addition to the basic k-means algorithm, SC3 (Kiselev* et al.* 2017) package utilizes a parallelization approach whereby a significant subset of the parameter space is evaluated simultaneously to obtain optimized clustering outcomes. In our practice, SC3 works well for low throughput scRNA-seq data and does not require heavy computing power, while unfit for the large scRNA-seq dataset. The main disadvantage of k-means algorithm-based approaches is that they are heavily dependent on the predetermined number of k, which often results in failure to detect the cell types of rare cell populations.

Graph clustering is an unsupervised clustering approach that involves a community detection-based algorithm. The Louvain (Blondel* et al.* 2008), a community detection-based algorithm, is currently one of the most popular methods used for single-cell data clustering. The Louvain method identifies distinct communities based on a nearest-neighbor network for the cells. The recently developed Leiden algorithm (Traag* et al.* 2019), which yields communities that are guaranteed to be connected, and is shown faster than the Louvain algorithm and uncovers better partitions. Both Louvain and Leiden algorithms were successfully adopted by Seurat and Scanpy, and been extensively used. The strength of these approaches is their speed, even for large datasets.

All these clustering approaches have a set of their own parameters that significantly affect the output and the corresponding biological interpretations. For k-means based methods, the pre-defined k value directly determined the cluster number. Similarly, when applying Louvain and Leiden to a K-Nearest Neighbour approach (KNN graph), the number of nearest neighbors also influences the clustering results, for example, if the nearest neighbors are set to 20, it is difficult to detect the cell groups with less than 20 cells. Besides, the Louvain and Leiden algorithm also has a resolution parameter that affects the size of clusters, with smaller resolutions detecting more clusters, and the clusters with smaller cell size. Unfortunately, there is no perfect rule for determining the optional parameters, the users must typically make their decisions based on their biological interpretations of the results with different parameter combinations. It is important that take into consideration of both computational and biological aspects of the datasets, for instance, one might calculate the rationality of the clusters by examining the cell types that are already known to exist in the given samples, and provides new biological clues for the *de novo* detected new cell types.

The most time-consuming step of scRNA-seq data analysis is biological interpretation and annotation of the cell clusters, and this step is tightly corelated to cell clusters. Generally, cell types are annotated by hand with well-known cell type specific marker genes, however, defining cell types based on only a few marker genes sometimes are arbitrary, and often it is inappropriate to draw comparison conclusions from different studies’ results directly, as they may use the different set of marker genes to define the “same” type of cells. For example, both CD14 and CD16 are monocytes markers, but they represent different subtypes of monocytes. Similarly, there are many different subtypes of endothelial cells while “endothelial cells” may be roughly labeled. Thanks to recent and ongoing efforts of the large single-cell database, such as the Human Cell Atlas or the mouse brain atlas, identifying and annotating cell types with the aid of external reference databases was applied, this greatly facilitated cell type annotation and was helpful to unify the definition across different studies.

There are two ways to annotate cell types with the aid of external reference datasets: using the data derived marker genes or using the full transcriptome profile. Differential expression testing is often an effective way to collect the marker genes: comparing the cells in one cluster to all other cells in the dataset. Typically, the marker genes have strong differential expression effects, and are easy to be detected with simple statistic tests such as *t*-test and Wilcoxon test, and the top-ranked genes are often considered as marker genes. Marker genes can be further loosely verified via visual inspection for their specificity. Clusters can be then annotated with the enrichment analysis by comparing the marker genes from the dataset and marker genes from the reference datasets. Recently, automated annotation tools, such as scmap (Kiselev* et al.* 2018) and Garnett (Pliner* et al.* 2019), are available for projecting cells from a scRNA-seq data set onto cell types or individual cells from other experiments by directly comparing the gene expression profile. These methods are easy to use and offer a vast increase in speed. However, if the reference datasets do not contain exactly the same cell type as the cells at the dataset under the survey, it is difficult for the methods to output reliable results. Thus, as discussed above, each has its own advantages and limitations for both manual and automated approaches, it is difficult to recommend which one is better than another. A combination of both may be the current best practice, to speed up this process, coarsely cell labels can be obtained by automated annotation first, and then manual annotation should be performed for verification. Remarkably, cell annotation has been detailing discussed in a recent study (Clarke* et al.* 2021).

## TRAJECTORY ANALYSIS

The heterogeneity of cell populations we observed from scRNA-seq data is often continues biological process during development *in vivo*. Thus, in order to capture transitions between cell identities in biological function, cell-trajectory analysis tools enable the temporal ordering of cell lineage in the notion of “pseudotime”. Currently, many cell-trajectory analysis tools have been developed based on dimension reduction, the nearest neighbor graph, cluster networks or RNA velocity algorithm in a supervised or unsupervised manner. A recent comprehensive comparison study benchmarked 45 trajectory analysis tools on a different dataset for cellular ordering, and highlight that the choice of method should depend mostly on the dataset dimensions and trajectory topology, and claim their guidelines (https://benchmark.dynverse.org) are helpful for users to select the best method for their dataset (Saelens* et al.* 2019). Our lab has tried Monocle (Trapnell* et al.* 2014), Harmony (Nowotschin* et al.* 2019), Palantir (Setty* et al.* 2019), PAGA (Wolf* et al.* 2019), Wishbone (Setty* et al.* 2016) and RNA velocity (La Manno* et al.* 2018), and they performed well on trajectory analysis, but often are dataset dependent (Guo* et al.* 2019; He* et al.* 2020; Yu* et al.* 2022). It is worth noting that, any inferred trajectory should be validated by an alternative method to avoid method bias.

## CELL–CELL COMMUNICATION

Deciphering cell–cell communication (CCC) from single-cell gene expression has been previously reviewed (Armingol* et al.* 2021). In brief, the signaling mediated by ligand–receptor, receptor–receptor and extracellular matrix-receptor interactions can be used to infer intercellular communication from the coordinated expression of their paired genes. CellPhoneDB (Efremova* et al.* 2020) is one of the earliest tools to infer CCC from the combined expression of multi-submit ligand–receptor complexes, which includes ~1400 known interactions from ~1000 proteins. CellChat (Jin* et al.* 2021) increases the interaction database to ~2000 ligand–receptor pairs, and incorporates signaling molecule interaction information from the KEGG Pathway database into their analysis. NichNet (Browaeys* et al.* 2020) predicts ligand–receptor links between cells, can predict which ligands influence the expression in another cell, which target genes are affected by each ligand and which signaling mediators may be involved. Deciphering CCC is helpful for us to understand cell development, tissue homeostasis and immune response. We previously uncovered interactions between different immune cell types, such as the interaction between monocytes/macrophages and T cells dysregulates in COVID-19 patients (Liu* et al.* 2020b; Ren* et al.* 2021). Although recently many new tools are developed, and more and more interactions are curated and added, it is still not a complete list of all possible ligand–receptor interactions for most tools. And CCC assessment will be significantly improved when combined with the spatial location of the cells from spatial omics.

## SOFTWARE

Software for scRNA-seq data analysis is rapid development in recent years. scRNA tools database (Zappia* et al.* 2018) (https://www.scrna-tools.org) and awesome-single-cell (https://github.com/seandavi/awesome-single-cell) collect a range of information on each scRNA-seq analysis tool and categorize them according to the analysis tasks they perform. Our lab uses the R package Seurat (Butler* et al.* 2018) and the python package Scanpy (Wolf* et al.* 2018), and found they give excellent and comparable results. Although the programming language of the tools is different, their hierarchical information for scRNA-seq data deposition is similar, and tools are developed to perform data transformation between platforms. SeuratDisk allows interoperability between Seurat and Scanpy. Our lab developed scDIOR (Feng* et al.* 2022) for single-cell data transformation between platforms of R and Python rapidly and stably, which is freely accessible at https://github.com/JiekaiLab/scDIOR. And we list some of the most popular tools we discussed above and their computational tasks in Table 1.

**Table 1:** The most commonly used tools for scRNA-seq data analysis

Name	Environment	URL
General purposes		
Seurat	R	satijalab.org/seurat/get_started.html
Scanpy	Python	https://scanpy.readthedocs.io/en/stable/
Pre-processing and quantification		
fastqc	Linux	https://www.bioinformatics.babraham.ac.uk/projects/fastqc/
Trimmomatic	Linux	http://www.usadellab.org/cms/?page=trimmomatic
Trim Galore	Linux	https://www.bioinformatics.babraham.ac.uk/projects/trim_galore/
cutadapt	Linux	https://cutadapt.readthedocs.io/en/stable/guide.html
RSEM	Linux	https://github.com/deweylab/RSEM
HTSeq	Linux	https://htseq.readthedocs.io/en/release_0.11.1/count.html
CellRanger	Linux	https://support.10xgenomics.com/single-cell-gene-expression/software/pipelines/latest/what-is-cell-ranger
STAR	Linux	https://github.com/alexdobin/STAR
scTE	Python	https://github.com/JiekaiLab/scTE
Quality control		
scrublet	Python	https://github.com/swolock/scrublet
DoubletFinder	R	https://github.com/chris-mcginnis-ucsf/DoubletFinder
scds	R	https://www.bioconductor.org/packages/release/bioc/html/scds.html
Normalization		
scran	R	https://bioconductor.org/packages/release/bioc/html/scran.html
sctransform	R	https://satijalab.org/seurat/articles/sctransform_vignette.html
Data correction		
fastMNN	R	http://bioconductor.org/packages/devel/bioc/vignettes/batchelor/inst/doc/correction.html
Scanorama	Python/R	https://github.com/brianhie/scanorama
Seurat V3	R	satijalab.org/seurat/get_started.html
LIGER	R	https://github.com/welch-lab/liger
Harmony	Python/R	https://github.com/immunogenomics/harmony
RPCI	R	https://github.com/bioinfoDZ/RISC
Feature selection		
scran	R	https://bioconductor.org/packages/release/bioc/html/scran.html
Seurat	R	satijalab.org/seurat/get_started.html
GiniClust	R	https://github.com/lanjiangboston/GiniClust
Dimensionality reduction and visualization		
t-SNE	Python/R	https://scikit-learn.org/stable/modules/generated/sklearn.manifold.TSNE.html; https://cran.r-project.org/web/packages/Rtsne/index.html
UMAP	Python/R	https://umap-learn.readthedocs.io/en/latest/index.html; https://github.com/jlmelville/uwot
Cluster analysis		
Louvain	Python	https://github.com/taynaud/python-louvain
Leiden	Python	https://github.com/vtraag/leidenalg
SC3	R	https://bioconductor.org/packages/release/bioc/html/SC3.html
Cell type annotation		
MetaNeighbor	R	https://github.com/maggiecrow/MetaNeighbor
CellAssign	R	https://github.com/Irrationone/cellassign
Garnett	R	https://cole-trapnell-lab.github.io/garnett/
Trajectory analysis		
Monocle	R	http://cole-trapnell-lab.github.io/monocle-release/
destiny	R	https://bioconductor.org/packages/release/bioc/html/destiny.html
PAGA	Python	https://scanpy.readthedocs.io/en/stable/
Palantir	Python	https://github.com/dpeerlab/Palantir
URD	R	https://github.com/farrellja/URD
Slingshot	R	https://www.bioconductor.org/packages/release/bioc/html/slingshot.html
scVelo	Python	https://scvelo.readthedocs.io/
Cell–cell communication		
CellPhoneDB	Python	https://github.com/Teichlab/cellphonedb
CellChat	R	https://github.com/sqjin/CellChat
NicheNet	R	https://github.com/saeyslab/nichenetr
Data transformation		
SeuratDisk	R	https://mojaveazure.github.io/seurat-disk/articles/convert-anndata.html
scDIOR	Python/R	https://github.com/JiekaiLab/scDIOR

## CONCLUSION

Computational analysis of scRNA-seq data developing rapidly, and it is foreseeable that there will be a vastly increasing number of analyzing tools for extracting the missing information from the data over the coming years. Particularly, we hope that there will be great improvements for the tools that provide integrated workflows, such as Seurat and Scanpy, making the analysis pipeline more comprehensive with intergradation of other omics analysis pipelines, and compatible with other commonly used analyzing tools.

## Conflict of interest

Jiangping He, Lihui Lin and Jiekai Chen declare that they have no conflict of interest.

## References

[bAnders2015] (2015). HTSeq - A Python framework to work with high-throughput sequencing data. Bioinformatics.

[bAndrews2021] (2021). Tutorial: guidelines for the computational analysis of single-cell RNA sequencing data. Nat Protoc.

[bArmingol2021] (2021). Deciphering cell-cell interactions and communication from gene expression. Nat Rev Genet.

[bBacher2016] (2016). Design and computational analysis of single-cell RNA-sequencing experiments. Genome Biol.

[bBais2020] (2020). scds: computational annotation of doublets in single-cell RNA sequencing data. Bioinformatics.

[bBaranGale2018] (2018). Experimental design for single-cell RNA sequencing. Brief Funct Genomics.

[bBarkas2019] (2019). Joint analysis of heterogeneous single-cell RNA-seq dataset collections. Nat Methods.

[bBecht2018] Becht E, McInnes L, Healy J, Dutertre CA, Kwok IWH, Ng LG, Ginhoux F, Newell EW (2018) Dimensionality reduction for visualizing single-cell data using UMAP. Nat Biotechnol. https://doi.org/10.1038/nbt.4314

[bBlondel2008] Blondel VD, Guillaume J-L, Lambiotte R, Lefebvre E (2008) Fast unfolding of communities in large networks. J Stat Mech10: P10008. https://doi.org/10.1088/1742-5468/2008/10/P10008

[bBolger2014] (2014). Trimmomatic: a flexible trimmer for Illumina sequence data. Bioinformatics.

[bBrennecke2013] (2013). Accounting for technical noise in single-cell RNA-seq experiments. Nat Methods.

[bBrowaeys2020] (2020). NicheNet: modeling intercellular communication by linking ligands to target genes. Nat Methods.

[bButler2018] (2018). Integrating single-cell transcriptomic data across different conditions, technologies, and species. Nat Biotechnol.

[bButtner2019] (2019). A test metric for assessing single-cell RNA-seq batch correction. Nat Methods.

[bCao2018] (2018). Joint profiling of chromatin accessibility and gene expression in thousands of single cells. Science.

[bClarke2021] (2021). Tutorial: guidelines for annotating single-cell transcriptomic maps using automated and manual methods. Nat Protoc.

[bCole2019] (2019). Performance assessment and selection of normalization procedures for single-cell RNA-Seq. Cell Syst.

[bCrow2018] (2018). Characterizing the replicability of cell types defined by single cell RNA-sequencing data using MetaNeighbor. Nat Commun.

[bDobin2013] (2013). STAR: ultrafast universal RNA-seq aligner. Bioinformatics.

[bEfremova2020] (2020). CellPhoneDB: inferring cell-cell communication from combined expression of multi-subunit ligand-receptor complexes. Nat Protoc.

[bFeng2022] (2022). scDIOR: single cell RNA-seq data IO software. BMC Bioinformatics.

[bGoke2015] (2015). Dynamic transcription of distinct classes of endogenous retroviral elements marks specific populations of early human embryonic cells. Cell Stem Cell.

[bGrindberg2013] (2013). RNA-sequencing from single nuclei. Proc Natl Acad Sci USA.

[bGuo2019] (2019). Resolving cell fate decisions during somatic cell reprogramming by single-cell RNA-Seq. Mol Cell.

[bHaghverdi2018] (2018). Batch effects in single-cell RNA-sequencing data are corrected by matching mutual nearest neighbors. Nat Biotechnol.

[bHainer2019] (2019). Profiling of pluripotency factors in single cells and early embryos. Cell.

[bHe2021] (2021). Identifying transposable element expression dynamics and heterogeneity during development at the single-cell level with a processing pipeline scTE. Nat Commun.

[bHe2020] (2020). Single-cell analysis reveals bronchoalveolar epithelial dysfunction in COVID-19 patients. Protein Cell.

[bHie2019] (2019). Efficient integration of heterogeneous single-cell transcriptomes using Scanorama. Nat Biotechnol.

[bJiang2016] (2016). GiniClust: detecting rare cell types from single-cell gene expression data with Gini index. Genome Biol.

[bJin2021] (2021). Inference and analysis of cell-cell communication using CellChat. Nat Commun.

[bKaminow2021] Kaminow B, Yunusov D, Dobin A (2021) STARsolo: accurate, fast and versatile mapping/quantification of single-cell and single-nucleus RNA-seq data. bioRxiv. https://doi.org/10.1101/2021.05.05.442755

[bKiselev2017] (2017). SC3: consensus clustering of single-cell RNA-seq data. Nat Methods.

[bKiselev2018] (2018). scmap: projection of single-cell RNA-seq data across data sets. Nat Methods.

[bKorsunsky2019] (2019). Fast, sensitive and accurate integration of single-cell data with Harmony. Nat Methods.

[bLa2018] (2018). RNA velocity of single cells. Nature.

[bLacar2016] (2016). Nuclear RNA-seq of single neurons reveals molecular signatures of activation. Nat Commun.

[bLafzi2018] (2018). Tutorial: guidelines for the experimental design of single-cell RNA sequencing studies. Nat Protoc.

[bLareau2020] (2020). Inference and effects of barcode multiplets in droplet-based single-cell assays. Nat Commun.

[bLeek2010] (2010). Tackling the widespread and critical impact of batch effects in high-throughput data. Nat Rev Genet.

[bLi2011] (2011). RSEM: accurate transcript quantification from RNA-Seq data with or without a reference genome. BMC Bioinformatics.

[bLin2019] (2019). scMerge leverages factor analysis, stable expression, and pseudoreplication to merge multiple single-cell RNA-seq datasets. Proc Natl Acad Sci USA.

[bLitvinukova2020] (2020). Cells of the adult human heart. Nature.

[bLiu2020a] (2020a). Jointly defining cell types from multiple single-cell datasets using LIGER. Nat Protoc.

[bLiu2020b] Liu X, Zhu A, He J, Chen Z, Liu L, Xu Y, Ye F, Feng H, Luo L, Cai B, Mai Y, Lin L, Zhang Z, Chen S, Shi J, Wen L, Wei Y, Zhuo J, Zhao Y, Li F, Wei X, Chen D, Zhang X, Zhong N, Huang Y, Liu H, Wang J, Xu X, Wang J, Chen R, Chen X, Zhong N, Zhao J, Li Y, Zhao J, Chen J (2020b) Single-cell analysis reveals macrophage-driven T cell dysfunction in severe COVID-19 patients. medRxiv. https://doi.org/10.1101/2020.05.23.20100024

[bLiu2021] (2021). Robust integration of multiple single-cell RNA sequencing datasets using a single reference space. Nat Biotechnol.

[bLuecken2019] (2019). Current best practices in single-cell RNA-seq analysis: a tutorial. Mol Syst Biol.

[bLun2016] (2016). Pooling across cells to normalize single-cell RNA sequencing data with many zero counts. Genome Biol.

[bMartin2011] (2011). Cutadapt removes adapter sequences from high-throughput sequencing reads. EMBnet J.

[bMcGinnis2019] (2019). DoubletFinder: doublet detection in single-cell RNA sequencing data using artificial nearest neighbors. Cell Syst.

[bMohammed2017] (2017). Single-cell landscape of transcriptional heterogeneity and cell fate decisions during mouse early gastrulation. Cell Rep.

[bNowotschin2019] (2019). The emergent landscape of the mouse gut endoderm at single-cell resolution. Nature.

[bPaik2020] (2020). Single-cell RNA sequencing in cardiovascular development, disease and medicine. Nat Rev Cardiol.

[bPapalexi2018] (2018). Single-cell RNA sequencing to explore immune cell heterogeneity. Nat Rev Immunol.

[bPliner2019] (2019). Supervised classification enables rapid annotation of cell atlases. Nat Methods.

[bPotter2018] (2018). Single-cell RNA sequencing for the study of development, physiology and disease. Nat Rev Nephrol.

[bRegev2017] (2017). The Human Cell Atlas. Elife.

[bRen2021] (2021). COVID-19 immune features revealed by a large-scale single-cell transcriptome atlas. Cell.

[bRisso2014] (2014). Normalization of RNA-seq data using factor analysis of control genes or samples. Nat Biotechnol.

[bRisso2018] (2018). A general and flexible method for signal extraction from single-cell RNA-seq data. Nat Commun.

[bSaelens2019] (2019). A comparison of single-cell trajectory inference methods. Nat Biotechnol.

[bSetty2019] (2019). Characterization of cell fate probabilities in single-cell data with Palantir. Nat Biotechnol.

[bSetty2016] (2016). Wishbone identifies bifurcating developmental trajectories from single-cell data. Nat Biotechnol.

[bStuart2019a] (2019). Comprehensive integration of single-cell data. Cell.

[bStuart2019] (2019). Integrative single-cell analysis. Nat Rev Genet.

[bTraag2019] (2019). From Louvain to Leiden: guaranteeing well-connected communities. Sci Rep.

[bTrapnell2014] (2014). The dynamics and regulators of cell fate decisions are revealed by pseudotemporal ordering of single cells. Nat Biotechnol.

[bvan2008] (2008). Viualizing data using t-SNE. J Mach Learn Res.

[bWang2019] (2019). CoBATCH for high-throughput single-cell epigenomic profiling. Mol Cell.

[bWolf2018] (2018). SCANPY: large-scale single-cell gene expression data analysis. Genome Biol.

[bWolf2019] (2019). PAGA: graph abstraction reconciles clustering with trajectory inference through a topology preserving map of single cells. Genome Biol.

[bWolock2019] (2019). Scrublet: computational identification of cell doublets in single-cell transcriptomic data. Cell Syst.

[bYu2022] (2022). BMP4 drives primed to naive transition through PGC-like state. Nat Commun.

[bZappia2018] (2018). Exploring the single-cell RNA-seq analysis landscape with the scRNA-tools database. PLoS Comput Biol.

[bZhang2019] (2019). Probabilistic cell-type assignment of single-cell RNA-seq for tumor microenvironment profiling. Nat Methods.

[bZheng2017] (2017). Massively parallel digital transcriptional profiling of single cells. Nat Commun.

